# Kinase signaling in the control of regulatory T cell function: molecular mechanisms and therapeutic implications

**DOI:** 10.3389/fimmu.2026.1857616

**Published:** 2026-07-15

**Authors:** Beibei Tang, Keke Li, Wanyu Chen, Juanjuan Xiao, Qiuhong Duan, Feng Zhu

**Affiliations:** 1 Translational Medicine Center, Huaihe Hospital of Henan University, Henan University, Kaifeng, China; 2 Henan International Joint Laboratory of Cell Medical Engineering, Huaihe Hospital of Henan University, Kaifeng, China; 3 Medical and Industry Crossover Research Institute of Medical College, Henan University, Kaifeng, China; 4 Department of Biochemistry and Molecular Biology, School of Basic Medicine, Huazhong University of Science and Technology, Wuhan, China

**Keywords:** inhibitors, kinases, molecular mechanisms, regulatory T cells, signaling pathways

## Abstract

Regulatory T cells (Tregs) are central to immune regulation, preventing excessive immune responses, maintaining immune tolerance, and modulating inflammatory microenvironments. Dysregulation of Treg development, differentiation, proliferation, or function contributes significantly to autoimmune diseases, inflammatory disorders, and tumor immune evasion. Protein kinases, key mediators of cellular signaling, regulate diverse processes including motility, metabolism, transport, and cell cycle progression; their emerging roles in immune regulation make them promising therapeutic targets for inflammatory diseases, autoimmunity, and immunotherapy-treated cancers. Notably, protein kinases modulate Treg differentiation and function by controlling the expression of the lineage markers Forkhead box protein 3 (Foxp3, intracellular) and CD25 (cell surface). This mechanistic review addresses: (1) fundamental Treg characteristics, functions, and disease relevance; (2) protein kinase-mediated regulatory mechanisms in Tregs; and (3) progress on protein kinase inhibitors for Treg-related diseases. The review aims to outline the kinase regulatory network governing Treg biology and guide future identification of kinase targets for treating autoimmune, inflammatory, and malignant diseases.

## 1 Introduction

Regulatory T cells (Tregs) are a specialized subset of T cells responsible for maintaining immune tolerance, suppressing excessive immune responses, and suppressing autoimmune diseases and chronic inflammation. Concurrently, Tregs exert immunosuppressive effects on both innate and adaptive anti-tumor immunity, thereby promoting tumor immune escape (1). Current Treg-based clinical strategies primarily encompass adoptive cell therapy and combination targeted drug therapy. Although adoptive cell therapy has demonstrated safety and efficacy in conditions such as kidney and liver transplantation, systemic lupus erythematosus, and inflammatory bowel disease, it faces limitations including short survival of cells *in vivo* and risks of systemic immunosuppression (2). Targeted drug combination therapy faces challenges such as immune-related adverse events, insufficient targeting specificity, the complexity of the tumor microenvironment (TME), and limited applicability (3).

Given the pivotal role of Tregs in immune regulation and the shortcomings of existing treatments, exploring novel regulatory mechanisms and developing innovative therapeutic strategies hold substantial scientific and clinical value for addressing major diseases such as cancer, inflammatory diseases, and autoimmune disorders. Protein kinases, as key regulators of Treg metabolism, signal transduction, and functional phenotype, represent important targets for precise modulation of Treg activity to enhance immunotherapeutic efficacy. The development of inhibitors targeting Treg-related protein kinases is thus of great significance for treating associated diseases.

This article provides a mechanistic review of the molecular characteristics of Tregs and their involvement in autoimmune diseases, inflammation, and tumors, with a focus on the regulatory mechanisms and intervention strategies involving protein kinases, aiming to provide potential therapeutic avenues for autoimmune, inflammatory, and malignant diseases.

## 2 Basic characteristics, classification, and associated diseases of regulatory T cells

Tregs play a vital role in maintaining immunological tolerance and inhibiting detrimental immune reactions. They encompass multiple subtypes, such as thymus-derived Tregs (tTregs) and peripherally derived Tregs (pTregs), with shared core features (e.g., Foxp3 expression) but distinct functional capacities (4). Their primary functions include suppressing effector T cell activation and maintaining immune homeostasis, and their loss or dysfunction can result in various autoimmune and inflammatory disorders (to be detailed in the following sections).

### 2.1 Basic characteristics of Tregs

Tregs are characterized by high expression of the IL-2 receptor α chain (IL-2Rα/CD25) (5). CD25 forms a heterotrimeric complex with IL-2Rβ (CD122) and IL-2Rγ (CD132). This complex increases receptor affinity and activates signaling pathways such as JAK/STAT5, PI3K/Akt/mTOR, and MAPK, thereby regulating cell proliferation, differentiation, and apoptosis (6–8). The transcription factor Foxp3 is another defining marker of Tregs, playing an essential role in their lineage stability, phenotype maintenance, and suppressive function (9, 10). However, Treg identification cannot rely solely on CD25 and Foxp3 expression, particularly in humans. It typically requires a combination of the CD25^+^Foxp3^+^CD127^low/–^phenotype, supplemented with *in vitro* suppression functional assays and analysis of demethylation at the Treg-specific demethylated region (TSDR) within the Foxp3 gene locus, to confirm lineage stability (10–12).

### 2.2 Classification and physiological functions of Tregs

Based on origin and function, Tregs fall into several subsets. These include thymus-derived Tregs (tTregs, also called natural Tregs or nTregs), peripheral Tregs (pTregs) that differentiate from antigen-stimulated T cells in peripheral tissues, and induced Tregs (iTregs) generated from naïve T cells by *in vitro* addition of TGF-β and IL-2 (13). Tregs can also be categorized by their activation state or tissue distribution into effector Tregs, tissue-resident Tregs, and tumor-infiltrating Tregs. pTregs modulate adaptive immune responses and help limit allergic inflammation at mucosal surfaces (14). iTregs maintain immune tolerance under severe inflammatory conditions and can support nTregs in restoring tolerance when needed (15). Effector Tregs show an activated phenotype (e.g., CD44^+^CD62L^-^) and act quickly to suppress effector T cells. Tissue-resident Tregs reside in non-lymphoid tissues such as adipose tissue, muscle, and skin, where they influence local immunity and metabolism. Tumor-infiltrating Tregs often express high levels of immune checkpoint molecules (e.g., CTLA-4, PD-1) and chemokine receptors (e.g., CCR4, CCR8), and are key players in tumor immune evasion (16). A clear set of lineage markers to distinguish tTregs from pTregs is currently lacking. Reliable identification usually requires a combined assessment of TCR lineage, epigenetic features (including Helios, Neuropilin-1 expression, and TSDR methylation status), and functional state (17, 18).

### 2.3 Associated diseases of Tregs

Abnormalities in the number or function of Tregs—which are key regulators of immune tolerance—can lead to autoimmune diseases, chronic inflammation, and immune evasion in various tumors.

#### 2.3.1 Tregs and autoimmune diseases

Autoimmune diseases result from broken immune tolerance, causing the body to attack its own tissues. Common examples include type 1 diabetes, systemic lupus erythematosus, rheumatoid arthritis, and multiple sclerosis. Tregs are essential for controlling aberrant immune responses, maintaining self-tolerance, and preventing excessive inflammation. Deficiencies in Treg number or function have been reported in most autoimmune diseases (19), underscoring their critical role in disease pathogenesis.

Type 1 diabetes (T1D) is an autoimmune disease characterized by the selective destruction of insulin-producing pancreatic β cells (20). In T1D patients, Tregs show reduced numbers, altered subset frequency, and abnormal expression of functional molecules such as PD-1, with Treg quality closely linked to the preservation of β cell function (21–24). The impaired suppressive function of Tregs leads to inadequate control of autoreactive T cells, resulting in autoimmune attack on pancreatic islet β cells—a key mechanism driving T1D onset and progression. Encouragingly, clinical and experimental studies have demonstrated that Treg-based therapy, or combination strategies that enhance Treg suppressive activity, can reduce islet damage and improve β cell function, suggesting that restoring Treg function represents a promising therapeutic strategy for T1D (25).

Systemic lupus erythematosus (SLE) is an autoimmune disorder characterized by autoantibodies against nuclear antigens. Circulating chromatin-autoantibody immune complexes deposit in multiple tissues, causing inflammation and tissue damage (26). One study in children with SLE reported that the percentages of CD4^+^CD25^high^Foxp3^+^ and CD8^+^CD25^high^Foxp3^+^ Tregs in peripheral blood increased following royal jelly supplementation, suggesting potential for Treg restoration (27). Additionally, an imbalance in the Th17/Treg ratio is commonly observed in SLE patients and is closely linked to a relative Treg deficiency (28). Beyond numerical defects, Tregs from SLE patients also show functional abnormalities, such as decreased IL-10 secretion, further disrupting immune tolerance (28). These collective defects impair Treg-mediated suppression of excessive effector T cell activation, thereby enhancing autoimmune responses and promoting SLE pathogenesis.

Rheumatoid arthritis (RA) is a systemic and heterogeneous autoimmune disorder characterized by symmetrical polyarthritis (29). An imbalance in the Th17/Treg ratio plays a critical role in RA pathogenesis. Th17 cells promote potent pro-inflammatory responses through IL-17 secretion, while dysfunctional Tregs fail to regulate autoreactive immune reactions (30). The impaired suppressive function of Tregs in RA manifests as decreased suppressive capacity and enhanced plasticity toward the Th17 phenotype. Even when enriched in synovial fluid, Tregs cannot effectively suppress local inflammation (31). Reduced expression of the Ikaros zinc finger transcription factor, leads to decreased Treg stability (32); IL-9 inhibits Treg production of IL-10 and TGF-β (33); Altered expression of chemokine receptors (e.g., CXCR3, CCR4, CCR5, CCR6) impairs Treg migration to inflamed joints in RA (34); additionally, certain synovial Tregs exacerbate inflammation by expressing ST2 and competitively consuming IL-33 (35). Clinically, low-dose IL-2 has been shown to specifically expand Tregs and restore the Th17/Treg balance, offering a promising therapeutic strategy for refractory RA (36).

Multiple sclerosis (MS) is a persistent autoimmune inflammatory condition affecting the central nervous system (CNS), leading to neuronal damage (37). MS pathogenesis is primarily driven by the activation of peripheral self-reactive effector CD4^+^ T cells, which infiltrate the CNS and trigger disease progression (38). Under normal conditions, specific Treg subsets—such as CD8^+^CD45RC ^low/neg^ Tregs—can migrate into the CNS and suppress neuroinflammation, thereby exerting a protective role (39). Treatment with fingolimod, an S1P receptor modulator, has been shown to increase the percentage of CD8^+^CD28^-^ regulatory T cells in MS patients, suggesting a potential mechanism for its therapeutic effect (40).

#### 2.3.2 Tregs and chronic inflammation

Defects in Treg proliferation and suppressive function are closely associated with the onset and progression of chronic inflammatory conditions such as psoriasis.

Psoriasis is a chronic inflammatory disease with strong genetic susceptibility, often triggered by environmental cues. In individuals with intact immune function, its pathogenesis is primarily mediated by type 1 and type 17 cytokine-producing cells, which are normally regulated by Treg (41). In psoriasis patients, Tregs exhibit significant functional abnormalities. Their impaired suppressive function leads to Th17/Treg imbalance, thereby exacerbating inflammation (41). Specifically, the functional defects of CD4^+^CD25^+^Foxp3^+^ Tregs are associated with IL-23-induced transformation into Th17 cells and with IL-17A-induced reduction of TGF-β1 and Foxp3 expression, which inhibits Treg activity (42). Additionally, circulating Tregs in psoriasis patients show impaired skin-homing ability. The numbers of CLA^hi^ and CLA^hi^CCR5^+^ Tregs are reduced, with decreased expression of CCR4 and CLA (cutaneous lymphocyte-associated antigen) and increased expression of CCR7. These changes result in insufficient suppression of inflammation in lesional skin (43). Currently, various treatments can ameliorate psoriasis by restoring Treg number and function or by modulating Treg-related pathways (42, 44).

#### 2.3.3 Tregs and tumors

Tumors encompass a broad category of diseases characterized by abnormal cell proliferation, with malignant tumors (cancer) posing the greatest threat to human health. The tumor microenvironment consists of non-tumor cells and extracellular components within the tumor niche, including bioactive molecules synthesized, secreted, or released by these cellular elements. Persistent bidirectional crosstalk between tumor cells and the TME critically regulates tumor initiation, progression, metastasis, and therapeutic response (45). Tregs promote immune escape, proliferation, and survival of tumor cells by suppressing innate and adaptive anti-tumor immune responses. Recent studies have elucidated several key mechanisms underlying Treg-mediated tumor immune escape (**Table 1**):

**Table 1:** Tregs and tumor immune evasion. Alt text: **Table 1** presents the key mechanisms by which Tregs mediate tumor immune escape.

Involved cells	Pathway	Mechanism
Tregs/effector T cells, APCs	Suppress effector T cells and APCs	Downregulate IL-2Rα on effector T cells or upregulate CD25 to compete for IL-2 (46, 47); secrete IL-10, IL-35, and TGF-β (49)
Tregs/NK cells, CD8^+^ T cells, DCs	Suppress the function of NK cells, CD8^+^ T cells, and DCs	Secrete perforin and granzyme B to induce apoptosis (52); bind checkpoint molecules (e.g., CTLA-4, PD-1) to block ligands on APCs (54)
Tregs	Recruit into TME	Migrate via CCR4 binding to tumor-secreted CCL22/CCL17 (60, 61)

1. Modulation of effector T cells via surface molecules. Tregs modulate effector T cells via surface molecules such as CTLA-4 and GITR, engaging target cells and thereby inhibiting IL-2Rα chain expression on effector T cells. Alternatively, Tregs upregulate CD25 to compete with effector T cells for IL-2 within the microenvironment, suppressing the activation and proliferation of effector T cells (CD4^+^CD25^+^CD127^low^Foxp3^+^. In lung cancer, changes in circulating CD4^+^CD25^+^CD127^low^Foxp3^+^ Treg numbers can predict responses to PD-1/PD-L1 inhibitors—their numbers increase in patients with hyperprogression, and decrease in responders, suggesting that Treg enrichment via IL-2 competition may hinder immunotherapy-induced effector T cell activation (48).
2. Secretion of immunosuppressive cytokines. Tregs produce IL-10, IL-35, and TGF-β, which suppress antigen-presenting cells (APCs) and effector T cells (49). IL-10 and TGF-β within the TME further promote clonal expansion of natural Tregs (nTregs) (50). In colorectal cancer, effector Tregs (the most immunosuppressive subset) are enriched in tumors and largely express VEGFR2. Their ability to secrete IL-10 and TGF-β1 can be inhibited by VEGFR2 inhibitors, indicating that VEGF-VEGFR2 signaling enhances the immunosuppressive cytokine-secreting capacity of effector Tregs (51).
3. Cytotoxic effects. Tregs directly kill NK cells and CD8^+^ T cells by secreting granzyme B (GzmB) and perforin, thereby suppressing anti-tumor immunity (52). In lung cancer, CCR8^+^ Tregs are enriched in tumors and inhibit CD8^+^ T cell GzmB expression and cytotoxicity which correlates with poor patient prognosis (53).
4. Regulation of dendritic cell (DC) function. Tregs express immune checkpoint molecules such as CTLA-4 and PD-1, which bind to ligands on APCs and suppress APC maturation and function (54). CTLA-4 binding to CD80/CD86 reduces their expression while upregulating PD-L1 expression (55–57). In lung cancer, abundant IFN-γ in tumor-draining mediastinal lymph nodes promotes Treg differentiation into Th1-like effector Tregs, which inhibit DC1-induced cytotoxic T cell activation via MHC II-dependent contact with type 1 conventional dendritic cells (DC1) (58). In myeloma patients, DCs promote Treg expansion through CD80/CD86-mediated interactions (59).
5. Directional migration into tumor tissue. Tregs bind to CCL22/CCL17 secreted by tumor cells via CCR4 receptors, enabling directed migration into the TME. The CCL5/CCR5 axis is also involved in Treg recruitment in pancreatic and colon cancers (60, 61). Colorectal cancer-associated Tregs exhibit a Th17-like phenotype with increased expression of the chemokine receptor GPR15 and secretion of IL-17/TNF-α, mediating Treg infiltration and suppressing CD8^+^ T cell-dependent anti-tumor immunity (62).

## 3 Summary of the regulatory mechanisms of protein kinases on Tregs

**Figure 1:**
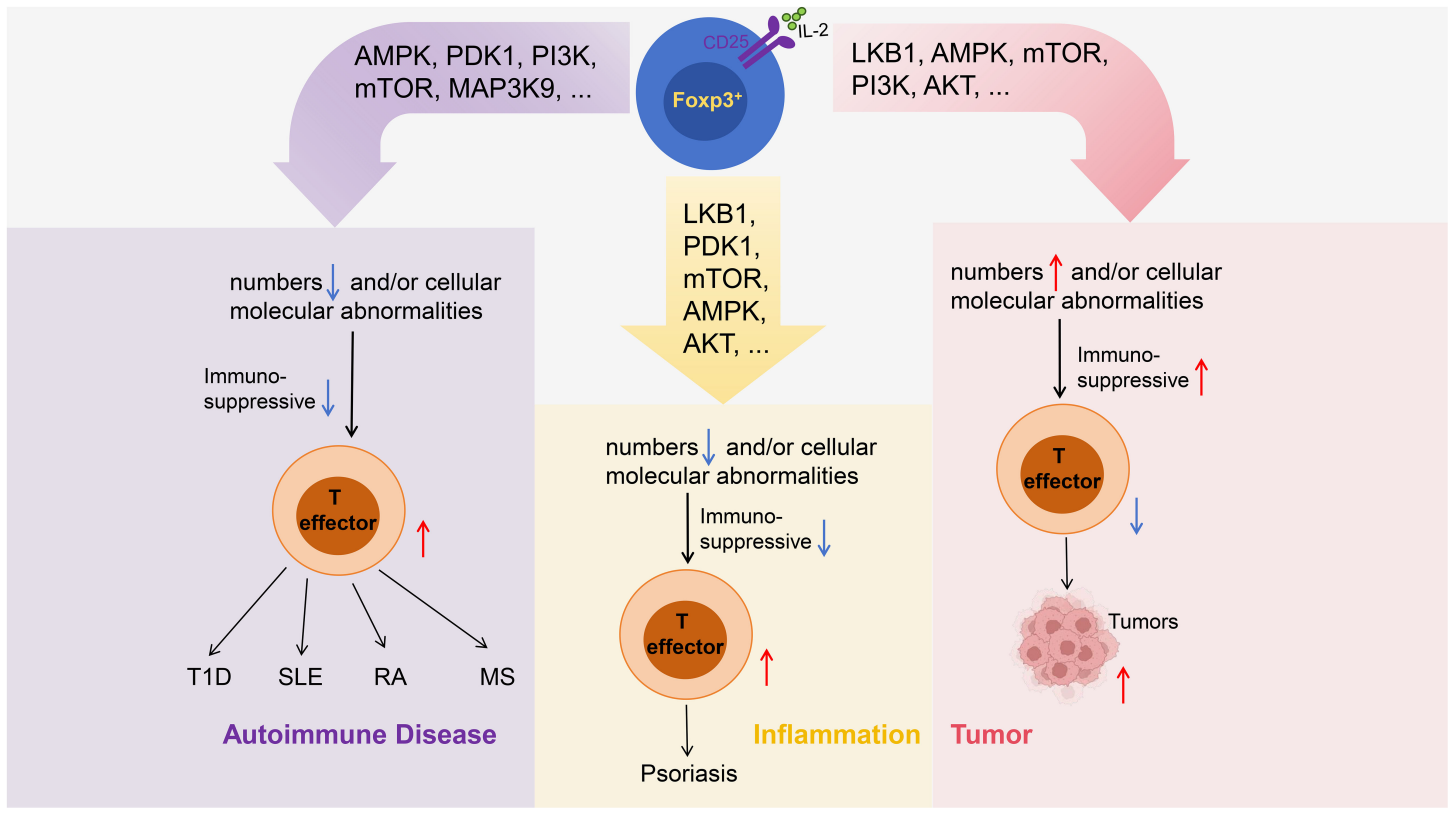
Kinase regulation of Tregs in disease pathogenesis. For a complete list, see **Table 2** and sections 4.1–4.3.

Regulatory T cells (Tregs) maintain immune tolerance and systemic homeostasis. Protein kinases regulate Treg differentiation, proliferation, function, the Th17/Treg balance, and Treg migration. **Figure 1** provides a concise summary of the key kinases involved in Treg regulation and their associations with disease pathogenesis. Detailed descriptions of each kinase and its molecular mechanism are provided in Sections 3.1-3.3 and **Table 2**.

**Table 2:** Protein kinases regulating Tregs: mechanisms, regulatory effects, and disease implications.

Kinase/Pathway	Upstream signals	Substrates/Mechanisms	Treg process regulation	Disease context	References
AKT1	PI3K	Phosphorylates Foxp3 at S418, reducing Foxp3 DNA-binding ability	Inhibits Foxp3 function	Human Treg cell model	(99)
AMPK	Dexmedetomidine; energy stress; TWEAK-HOIP axis	Phosphorylates Foxp3 to inhibit ubiquitination; downregulates the expression of E3 ubiquitin ligase STUB1, inhibits ubiquitination, and proteasomal degradation of Foxp3; upregulates Foxp3 expression; induces demethylation of mitochondrial gene promoters; and reduces CD25 expression	Differentiation, Foxp3 stability, suppressive function, and metabolic adaptation	Sepsis-related ARDS, autoimmune liver disease, melanoma, HCC, aged EAE; AMPKα2 promotes immune escape in HCC	(70, 89–93, 101)
AMPK/SIRT1	Melatonin	Enhances SIRT1 expression and stability	Restores the Th17/Treg balance	intestinal inflammation	(109)
CDK2	/	Phosphorylates Foxp3 at S19, S88, T114, T175	Negatively regulates Foxp3 expression, downregulates Treg function	Murine autoimmune disease model	(98)
CDK8/19	/	Inhibition of CDK8/19 induces Foxp3 expression	Promotes conversion of effector/memory T cells to Tregs	/	(103)
CK2	/	Enhances STAT3 phosphorylation, inhibits STAT5 phosphorylation	Enhances Th17 differentiation; inhibits Treg generation	Murine colitis model; CK2 inhibitors have therapeutic potential	(104)
GCN2K	IDO-mediated tryptophan deficiency	Inhibits T cell (including Treg) proliferation	Enhances Treg functional tolerance	Tumor immune microenvironment, transplant tolerance	(115)
HPK1	TCR signaling	Negatively regulates the TCR signaling pathway	Indirectly affects Treg function	Autoimmune diseases, tumor immunity	(74)
ITK	TCR signaling	Regulates Foxo1 nuclear translocation	Negatively regulates Foxp3 expression, affects Th17/Treg balance	Rheumatoid arthritis; ITK inhibitors increase Treg proportion	(112, 114)
LKB1	Energy/polarity signals; tumor-derived AGR2	Inhibits STAT4-mediated CpG methylation at Foxp3 CNS2; regulates the dendritic cell ARG1 axis	tTregs development, Foxp3 stability, Treg lineage stability, immune microenvironment modulation	aGVHD, tumor immune microenvironment; potential therapeutic target	(94–96)
MAP4K3 (GLK)	PKCθ-independent	Phosphorylates IKKβ (Ser733) → FoxO1 (Ser319) phosphorylation and nuclear translocation	Reduces Foxp3 expression, impairs Treg function	Autoimmune diseases	(73)
Mst1/Mst2	IL-2 signaling	Activates IL-2-STAT5 signaling, maintains CD25 and Foxp3 expression	Maintains the Treg population and lineage stability	Autoimmune diseases	(75)
mTOR	Nutrient/growth factors; TGF-β; TLR8	Regulates mTORC1/mTORC2; modulates glycolysis, mitochondrial function, GzmB expression; activates Stat3/inhibits Stat5; regulates CD62L, CCR7, S1P1, CD69	Differentiation, proliferation, function, stability, Th17/Treg balance, and migration to non-lymphoid tissues	Hepatic ischemia-reperfusion injury, sarcoidosis, ovarian cancer, kidney injury, EAE; rapamycin promotes Treg expansion; AZD8055 induces Treg dysfunction	(76–86, 106– 107, 120)
NLK	/	Phosphorylates Foxp3 at S19, S156, S189, S273, S278, S295, T341, preventing polyubiquitination	Enhances Foxp3 stability and maintains Treg function	Autoimmune diseases, tumor immunotherapy targets	(71)
PDK1	Downstream of PI3K	PDK1-MEK-ERK-CD71 axis; regulates ROS and iron metabolism	Treg survival, function, and number	Systemic autoimmunity, multi-tissue inflammatory damage	(87, 88)
PI3K/Akt/FoxO1	Exenatide; metabolic signals	Regulates RORγt vs. Foxp3 expression	Regulates Th17/Treg balance	Diabetic obesity, inflammatory diseases	(110)
PIM1	/	Phosphorylates Foxp3 at S422, reducing DNA-binding ability	Downregulates Foxp3 target genes, inhibiting Treg function	Autoimmune diseases, tumors	(10)
PIM2	/	Phosphorylates Foxp3 at S33, S41, S52, T56, S57, S58, and S59	Inhibits Foxp3 target gene expression, and downregulates Treg function	Autoimmune diseases, tumors	(9)
PKA	Buserelin	Activates the PKA pathway → downregulates Foxp3, upregulates RORγt and IL-17	Promotes Treg-to-Th17 polarization	Central precocious puberty	(113)
STK4	/	Phosphorylates Foxp3 at S418, enhancing transcriptional activity	Enhances Foxp3 expression and promotes Treg function	Autoimmune diseases, tumor immunity	(97)
TAK1	/	Maintains Treg survival (knockout induces apoptosis)	Treg survival	Autoimmune diseases	(72)

Summarizes the regulatory effects and mechanisms of the major protein kinases mentioned in this paper on Tregs.

**Figure 2:**
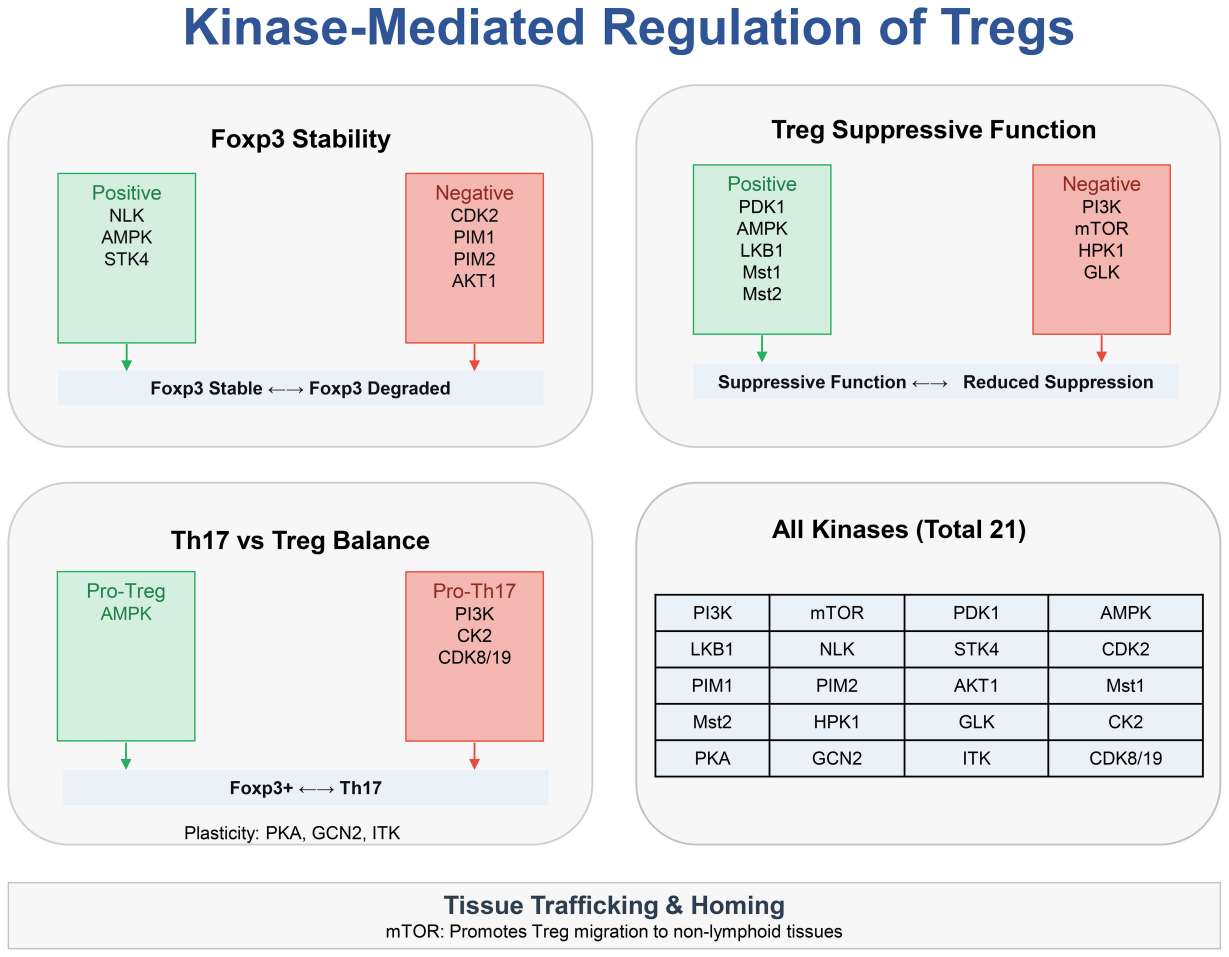
Protein kinases involved in the biological effects of Tregs.

Protein kinases catalyze protein phosphorylation and play essential roles in cellular activities. Abnormal expression or mutations of these kinases are closely associated with tumorigenesis and inflammation (63). By modulating immune cell signal transduction, protein kinases mediate the phosphorylation of approximately one-third of human proteins and contribute to diseases such as psoriasis, rheumatoid arthritis (RA), and systemic lupus erythematosus (SLE) (64–67). Thus, elucidating their molecular mechanisms is of considerable scientific importance. **Figure 2** provides a detailed summary of all the protein kinases mentioned in this article that are involved in Treg regulation.

### 3.1 Kinase-mediated regulation of regulatory T cell homeostasis and immunosuppressive function

Kinases regulate Treg biology at multiple levels, forming an integrated network that controls Treg functional integrity, lineage stability, and immunosuppressive capacity (68, 69). Treg homeostasis—defined as the maintenance of appropriate cell number, normal suppressive function, and stable lineage identity—is essential for immune balance. This section discusses three interconnected layers of kinase-mediated regulation. First, metabolic sensing kinases (PI3K, mTOR, PDK1, AMPK, and LKB1) coordinate Treg proliferation, survival, and metabolic adaptation (Section 4.1.1; 69). Second, kinases directly modulate Foxp3 stability and transcriptional activity through opposing phosphorylation events (Section 4.1.2; 68, 70, 71). Third, additional kinases at the TCR signaling interface or in specialized contexts fine-tune Treg development and function (Section 4.1.3; 72–75). Together, these regulatory layers ensure proper Treg homeostasis, and their dysregulation contributes to inflammatory diseases, autoimmunity, and cancer (63–65).

#### 3.1.1 Kinase-mediated regulation of Treg functional integrity and lineage stability via metabolic pathways

Whether a Treg maintains or loses its suppressive identity depends directly on its metabolic state. Nutrient, oxygen, and growth factor signals are integrated by several metabolic sensing kinases—AMPK, LKB1, PI3K, PDK1, and mTOR (69). These kinases coordinate Treg proliferation, survival, and lineage stability. Rather than acting independently, these pathways form an interconnected network that adapts Treg function to the metabolic demands of different tissue environments.

The PI3K-AKT-mTOR axis serves as the central metabolic rheostat, with PI3K signaling exerting context-dependent effects on Tregs. This pathway governs cellular growth and metabolism via the PI3K/Akt/mTOR cascade. During Treg induction, the acidic microenvironment resulting from hepatic ischemia-reperfusion injury (HIRI) suppresses the differentiation and function of CD4^+^CD25^+^Foxp3^+^ iTregs through this signaling axis. Restoring physiological pH rescues Foxp3 expression and iTreg function (76). In inflammatory settings such as pulmonary sarcoidosis models, pharmacological inhibition of overactive PI3K/Akt signaling in Tregs indirectly enhances their suppressive capacity (77). Selective inhibition of PI3Kγ/δ restores Treg immunosuppressive function and alleviates pulmonary granuloma pathology in sarcoidosis models (78). Under homeostatic conditions, distinct PI3K isoforms play differential roles in maintaining mature Tregs. Notably, PI3Kδ is a key regulator of Treg function. Stark et al. (79) showed that while PI3Kδ deficiency alone impairs Treg function without provoking autoimmunity, combined loss of PI3Kα and PI3Kδ markedly aggravates experimental autoimmune encephalomyelitis (EAE), indicating that multiple PI3K isoforms act together to preserve Treg stability under inflammatory stress. Thus, PI3K signaling in Tregs is not governed by a unitary regulatory logic; rather, its functional impact depends on both the pathological context and the specific isoforms involved. The requirement for concurrent loss of PI3Kα and PI3Kδ to impair Treg function indicates that isoform redundancy within this pathway provides a safeguard against environmental fluctuations.

mTOR functions through two distinct complexes, mTORC1 and mTORC2. Although this kinase belongs to the phosphatidylinositol kinase-related kinase (PIKK) family, it is classified as a non-classical serine/threonine protein kinase. During Treg induction and differentiation, inhibiting mTORC1 is critical for pTreg maturation. TGF-β-driven transcriptional reprogramming combined with mTORC1 downregulation promotes Treg differentiation via the DAP5/eIF3d non-classical translation mechanism (80). Moderate mTORC1 activation supports naïve T cell differentiation into iTregs and enhances their suppressive function, whereas persistent hyperactivation antagonizes Foxp3 expression and impedes Treg induction (81). Under steady-state conditions, mTORC1 positively regulates mature Tregs, helping maintain cellular stability and immunosuppressive function. mTORC1 deficiency reduces Treg stability and suppressive capacity, leading to autoimmune disease (82). Xiang et al. (83) showed that excessive mTORC1 activation markedly increases glycolysis in Tregs, reduces Foxp3 expression, directly compromises Treg stability, promotes transformation into effector T cells, and ultimately results in loss of immunosuppressive function in inflammatory environments. Rapamycin, a classical mTORC1 inhibitor, promotes selective expansion of functionally mature Tregs, reduces granzyme B expression and activity, and decreases S6K and c-Jun phosphorylation, thereby inhibiting Treg apoptosis (84). In the ovarian cancer microenvironment, TLR8 signaling suppresses glycolysis in CD4^+^ Tregs by downregulating mTOR activity, reversing their immunosuppressive function (85). The dual mTORC1/C2 inhibitor AZD8055 induces mitochondrial stress, autophagic defects, and energy metabolism disorders via a distinct mechanism, ultimately causing loss of proliferative potential and suppressive function in Tregs (86). These findings collectively suggest that mTORC1 activity in Tregs must be maintained within a relatively narrow range. Insufficient mTORC1 signaling compromises Treg functional integrity, whereas excessive signaling promotes glycolytic metabolism and suppresses Foxp3 expression. Thus, mTOR does not function as a simple positive or negative regulator of Treg activity; rather, it defines a permissive metabolic window, outside of which Treg function deteriorates.

PDK1, a key effector downstream of PI3K, is essential for Treg survival and function. Hyunju Oh et al. (87) generated a Treg-specific conditional PDK1 knockout mouse model and demonstrated that PDK1 deficiency directly impairs Treg function, leading to fatal systemic inflammatory responses resembling Scurfy mice. Previous studies indicate that PDK1 regulates redox homeostasis to support Treg survival. PDK1-deficient Tregs show excessive accumulation of reactive oxygen species (ROS), likely via the PDK1-MEK-ERK-CD71 signaling axis. Elevated ROS disrupts iron metabolism in Tregs, causing iron overload, which in turn reduces Treg numbers and impairs their function, ultimately resulting in fatal systemic autoimmunity and multi-tissue inflammatory damage (88). In conclusion, PDK1 functions beyond signal transduction by directly coupling PI3K signaling with redox homeostasis and iron metabolism. This positions PDK1 as a critical node in the kinase network, where its loss does not merely impair Treg function but triggers cell death through oxidative stress.

AMPK functions as a cellular energy sensor that helps Tregs adapt to metabolic stress. In sepsis-related ARDS, dexmedetomidine-induced AMPK/SIRT1 activation during Treg differentiation promotes the commitment of naïve CD4^+^ T cells to the Treg lineage, upregulates Foxp3 and Helios expression, and reduces lung inflammation (89). In an autoimmune liver disease model, AMPK enhances Foxp3 protein stability by phosphorylating Foxp3 and inhibiting its ubiquitination, thereby alleviating disease symptoms. Systemic or Treg-specific deletion of AMPKα1 impairs Treg suppressive function without affecting their proliferation (90). The AMPK catalytic subunit α exists as two isoforms, α1 and α2. In a mouse melanoma model with Treg-specific conditional knockout of AMPKα, the two isoforms were found to differentially regulate Treg suppressive function in the TME: AMPKα1 interacts with DNMT1 to promote demethylation of mitochondrial gene promoters in tumor-infiltrating Tregs, enhancing mitochondrial metabolism to help these cells cope with microenvironmental stress; AMPKα2 directly regulates Treg inhibitory function (91). As Ouyang et al. (92) further revealed, AMPKα2 (encoded by PRKAA2) promotes CD8^+^ T cell exhaustion and contributes to Treg formation in hepatocellular carcinoma. At the post-translational level, beyond classical phosphorylation regulation, the recently identified TWEAK-HOIP axis can directly induce AMPK inactivation through linear ubiquitination, driving metabolic reprogramming and abnormal cell proliferation (93). AMPK exerts dual effects in Tregs: its α1 isoform modulates mitochondrial metabolism through epigenetic remodeling, while α2 controls suppressive function, and both directly stabilize Foxp3 via phosphorylation. AMPK thus antagonizes mTOR while simultaneously uncoupling Foxp3 stability from metabolic flux—a feature that sustains Treg identity during energy stress.

LKB1, a serine/threonine kinase transcribed from the STK11 gene, is involved in regulating energy metabolism, cell growth, and cell polarity. In dendritic cells (DCs), LKB1 serves as a critical brake on DC immunogenicity. Loss of LKB1 in DCs indirectly promotes the development and expansion of tTregs (94). Recent findings indicate that LKB1-deficient tumor cells secrete AGR2, which impairs the intrinsic ARG1 axis in dendritic cells and thereby indirectly modulates the immune microenvironment where Tregs reside (95). In acute graft-versus-host disease (aGVHD), LKB1 within Tregs directly maintains stable Foxp3 expression and Treg lineage identity by inhibiting STAT4-mediated methylation of CpG sites in the conserved non-coding sequence 2 (CNS2) of the Foxp3 locus, thus preserving Treg stability and suppressive function (96). These findings suggest that LKB1-related pathways are potential therapeutic targets. Unlike AMPK and mTOR, which regulate metabolism and protein stability, LKB1 governs Treg epigenetic identity by maintaining CNS2 demethylation and preserving the transcriptional program. LKB1 deficiency thus imposes a permanent epigenetic defect rather than a reversible metabolic impairment.

These kinases form an integrated network with redundancy, division of labor, and hierarchical control. PI3K-mTOR sets the threshold for metabolic plasticity, PDK1 provides essential survival signals, AMPK couples energy status to Foxp3, and LKB1 preserves epigenetic integrity. Because the network is adaptive, targeting individual kinases produces context-dependent effects. Thus, stabilizing Tregs in disease requires understanding how the entire network interprets metabolic conditions to maintain or lose Treg identity.

#### 3.1.2 Kinase-mediated phosphorylation and ubiquitination of Foxp3 in Tregs

Foxp3 post-translational regulation is governed by a balance among multiple kinase networks. These kinases converge on Foxp3 to modulate its stability, with the net outcome—whether suppression, plasticity, or functional loss—reflecting the relative activities of competing nodes within this signaling network.

Foxp3 protein stability and transcriptional activity are dynamically controlled by post-translational modifications. Phosphorylation and ubiquitination work in opposition or in sequence to determine how much functional Foxp3 a Treg expresses at any given time. Multiple kinases converge directly on Foxp3, and the balance between stabilizing and destabilizing modifications defines Treg suppressive capacity (68).

Kinases that stabilize Foxp3: NLK stabilizes Foxp3 by phosphorylating residues S19, S156, S189, S273, S278, S295, and T341, which inhibits polyubiquitination and supports Treg function (71). AMPK also enhances Foxp3 stability by reducing CHIP-mediated ubiquitination (70). STK4 promotes Foxp3 transcriptional activity via phosphorylation at S418 (97).

Kinases that destabilize Foxp3: CDK2 negatively regulates Foxp3 expression and impairs Treg function by phosphorylating the S19, S88, T114, and T175 sites of Foxp3 (98). PIM1 negatively regulates the DNA-binding ability of Foxp3 by phosphorylating the S422 site of Foxp3, downregulating the expression of its target genes (10). PIM2 negatively regulates Foxp3 expression and inhibits the expression of its downstream target genes by phosphorylating the S33, S41, S52, T56, S57, S58, and S59 sites of Foxp3 (9). AKT1 reduces the DNA-binding ability of Foxp3 by phosphorylating the S418 site of Foxp3 (99).

The IL-2/CD25/STAT5 signaling axis provides an upstream control point for Foxp3 expression. In terms of direct cell-cell contact, besides suppressive signals mediated by molecules such as GITR (glucocorticoid-induced TNFR-related protein) and LAG-3 (lymphocyte activation gene 3), IL-2 can also enhance Treg suppressive function through non-classical pathways involving CD25-chemokine receptor complexes, which activate downstream JAK-STAT5 signaling (100). This pathway is critical for Treg maintenance. In aged EAE mice, AMPK deficiency in Tregs impairs IL-2-STAT5 signaling, reduces Foxp3 expression, and compromises the suppression of inflammation (101). Mst1/Mst2 (mammalian sterile 20-like kinase 1/2) kinases maintain Treg homeostasis by sensing IL-2 signals to activate STAT5; Mst1 deficiency leads to decreased expression of CD25 and Foxp3 in Tregs after IL-2 stimulation (75).

Ubiquitination as a counterbalance: The E3 ligase CHIP (STUB1) targets Foxp3 for degradation. AMPKα1 reduces CHIP expression, thereby protecting Foxp3 (70).

At these sites, a functional dichotomy emerges: stabilizing phosphorylation events block ubiquitination, whereas destabilizing phosphorylation either promotes proteasomal degradation or disrupts Foxp3 DNA binding. Thus, site-specific phosphorylation serves as a reversible post-translational switch that controls Foxp3 stability and transcriptional activity independently of Foxp3 expression levels.

#### 3.1.3 Kinase-mediated regulation of Treg development and functional modulation

Beyond the major metabolic and post-translational regulators, several other kinases participate in Treg biology, though their mechanisms are less well understood. These kinases often act at the TCR signaling interface or in specialized tissue contexts.

TCR-proximal kinases: Hematopoietic progenitor kinase 1 (HPK1), a Ste20 family kinase specifically expressed in hematopoietic cells, negatively regulates TCR signaling and thereby influences Treg function (74). MAP4K3 (GLK) phosphorylates IKKβ at Ser733 in a PKCθ-independent manner, leading to FoxO1 phosphorylation at Ser319 and its subsequent nuclear translocation, which in turn represses Foxp3 expression and impairs Treg suppressive function. This pathway thus illustrates a direct link between kinase activity and the acquisition of a pathogenic Treg phenotype in autoimmune disease (73). TAK1 is essential for Treg survival; conditional knockout of TAK1 in mouse Tregs induces apoptosis (72). Thus, these TCR-proximal kinases demonstrate that the intensity of TCR signaling influences not only Treg survival but also their functional stability.

Transcriptional and cell-cycle-associated kinases: As a target of miR-20a, MAP3K9 suppresses Treg differentiation in the EAE model. By downregulating MAP3K9, miR-20a inhibits the differentiation of antigen-specific CD4^+^ T cells into Tregs, indicating its potential for treating EAE and central nervous system demyelinating disorders (102). Chemical inhibition or genetic knockout of CDK8/19 induces Foxp3 expression and promotes the conversion of effector/memory T cells into Foxp3^+^ Tregs (103). Thus, cell-cycle kinases both stabilize established Treg phenotypes and reprogram effector cells toward a Treg fate, suggesting these kinases as nodes for therapeutic modulation of Treg plasticity. PIP4K similarly supports Treg signaling and suppressive function, reinforcing a recurring principle: kinases couple metabolic input to effector output in Tregs.

Kinases affecting Treg development: Mst1 and Mst2 (discussed in 3.1.2) maintain Treg homeostasis by sensing IL-2 signals. Their deficiency reduces CD25 and Foxp3 expression following IL-2 stimulation (75).

### 3.2 Kinase-directed regulation of the Th17/Treg axis *in vivo*

Kinases regulate the Th17/Treg axis at two levels. Section 4.2.1 describes how kinases direct naïve CD4^+^ T cell lineage commitment via mTOR/STAT3/STAT5, PI3K/Akt/FoxO1, AMPK/SIRT1, and CK2 signaling (104–110). Section 4.2.2 examines how kinases control Treg-to-Th17 plasticity under inflammatory conditions through STAT6, PKA, GCN2, and ITK (111–115). Together, these mechanisms maintain the dynamic balance of the Th17/Treg axis, and their disruption drives inflammatory and autoimmune diseases.

#### 3.2.1 Kinase-directed regulation of Th17/Treg lineage commitment via STAT3/STAT5 and FoxO1 signaling

The balance between Th17 and Treg cells is set during the differentiation of naive CD4^+^ T cells and is dynamically adjusted by cytokines and metabolic signals. Kinases influence where naïve CD4^+^ T cells end up—Th17 or Treg—via three broadly conserved mechanisms: they modulate phosphorylation of signaling intermediates (STAT3 vs. STAT5 being a textbook example), they regulate Foxp3 expression levels and stability in Tregs, and they control FoxO1 subcellular localization, which in turn sets the balance between Treg generation and Th17 differentiation. Below, we discuss how mTOR, PI3K/Akt, AMPK/SIRT1, and CK2 operate not as independent pathways but as interconnected nodes within a unified regulatory network.

mTOR as a master integrator: The mTOR pathway provides the clearest example. Jiao et al. (106) demonstrated that mTOR signaling regulates T cell differentiation by activating Stat3 and inhibiting Stat5: activated Stat3 induces RORγt and IL-17 expression, promoting Th17 differentiation; whereas activated Stat5 upregulates Foxp3, supporting Treg differentiation. In a kidney injury model, disruption of mTOR signaling reduces RORγt and increases Foxp3 expression; mesenchymal stem cells (MSCs) suppress Th17 differentiation from CD4^+^ T cells and promote Treg generation, thereby inhibiting T cell proliferation and alleviating inflammation (107). Luo et al. (108) further confirmed that during naive CD4^+^ T cell differentiation, MSCs reduce HIF-1α expression and enhance Stat5 phosphorylation by interfering with mTOR phosphorylation, thereby upregulating Foxp3 and promoting Treg generation. Of note, mTOR blockade leads to pronounced thymic involution and reduced T cell output. During Treg induction, disruption of mTORC1 signaling biases naive T cells toward Treg differentiation, whereas the mTORC2-Akt-FoxO1/3a pathway promotes conventional T cell activation, thereby indirectly limiting Treg differentiation (105).

PI3K/Akt/FoxO1 as a shuttling rheostat: The PI3K/Akt/FoxO1 pathway also influences lineage decisions. This signaling cascade is critical for T cell development, and its dysfunction is closely associated with various inflammatory diseases. It regulates inflammation by modulating the balance between Th17 and Tregs. Notably, inhibition of RORγt-mediated Th17 differentiation produces marked anti-inflammatory effects. In a diabetic obese mouse model, exenatide was shown to regulate the Th17/Treg balance via the PI3K/Akt/FoxO1 pathway (110).

AMPK/SIRT1 as a metabolic checkpoint: AMPK/SIRT1 signaling is also involved. In a mouse model of necrotizing enterocolitis (NEC), SIRT1 regulates the differentiation and function of CD4^+^ T cell subsets. Melatonin activates the AMPK/SIRT1 pathway to enhance SIRT1 expression and stability, thereby restoring the Th17/Treg balance and alleviating intestinal inflammation (109).

CK2 as a dual regulator: CK2 signaling is also involved. The conserved serine/threonine kinase CK2, widely expressed in eukaryotic cells, regulates cell proliferation, apoptosis, and inflammatory responses. In the TNBS-induced mouse colitis model, CK2 inhibitors suppress Th17 differentiation and promote Treg generation by reducing STAT3 phosphorylation while increasing STAT5 phosphorylation (104).

In conclusion, Th17/Treg differentiation is governed not by any single kinase but by an integrated phosphorylation network. mTOR, PI3K/Akt, AMPK, and CK2 converge on three downstream effectors—STAT3/STAT5 phosphorylation balance, FoxO1 nuclear localization, and Foxp3 expression—whose combined output sets the Th17/Treg equilibrium. Disruption of this integrated logic, rather than alteration of any individual kinase, is what drives inflammatory pathology.

#### 3.2.2 Kinase-directed regulation of Treg-to-Th17 plasticity *in vivo*

Tregs are not terminally differentiated. Under inflammatory conditions, some Tregs lose Foxp3 expression and convert into Th17-like cells (exTreg-Th17). However, enumerating the kinases involved in this conversion is insufficient to explain how they coordinately control Treg stability, metabolism, and functional plasticity. A more productive approach is to integrate these observations into a coherent signaling framework.

Kinases that promote Treg-to-Th17 conversion: Under IL-6-rich conditions, a subset of Tregs with unstable Foxp3 expression can differentiate into Th17-like cells (exTreg-Th17). Activation of STAT6 promotes this conversion by further destabilizing Foxp3 and reducing the suppressive function of Tregs. Conversely, STAT6-deficient iTregs maintain high Foxp3 and CD25 expression upon IL-6 stimulation. As STAT6 acts downstream of JAK kinases, this suggests a functional link between JAK-STAT6 signaling and IL-6-driven Treg instability (111).

Protein kinase A (PKA) also promotes plasticity. In a rat model of central precocious puberty (CPP), buserelin downregulates Foxp3 expression by activating the PKA pathway, while simultaneously promoting RORγt and IL-17 expression in Tregs, which may lead to their conversion into pro-inflammatory Th17 cells (113). Thus, a single kinase pathway can both promote and restrain the same Treg fate—depending on context—rather than exerting a unidirectional effect.

GCN2 (general control nonderepressible 2) kinase can sense the state of amino acid deficiency; it is activated in the tryptophan-deficient microenvironment (such as under IDO-mediated conditions), thereby inhibiting the proliferation of T cells (including Tregs) and enhancing their functional tolerance (115). Unlike STAT6 or PKA, GCN2 does not directly induce Foxp3 loss or Th17 polarization. Instead, it limits Treg proliferation and metabolic activity, rendering them vulnerable to functional instability under nutrient-restricted conditions. Thus, GCN2 acts by constraining Treg fitness rather than by dictating fate directly.

ITK belongs to the Tec kinase family and plays an important role in TCR signal transduction and Th cell differentiation. Studies have found that ITK can negatively regulate the expression of Foxp3 in CD4^+^ T cells, affecting the Th17/Treg balance (114). Among patients with RA, inhibition of ITK activity not only interferes with the generation of follicular helper T cells (Tfh) but also, by regulating FoxO1 nuclear translocation, effectively rebalances Th17 and Treg cells, thereby reducing Th17 and Th1 proportions while increasing the proportion of Foxp3^+^ Tregs (112). Thus, ITK not only promotes Treg plasticity but also couples FoxO1 localization to lineage stability. High ITK activity destabilizes Tregs, whereas moderate inhibition restores their functional equilibrium.

These kinases form a three-layered collaborative network rather than functioning in isolation. STAT6 and PKA regulate transcriptional determinants of cell fate, GCN2 sets the metabolic threshold permissive for plasticity, and ITK couples TCR signaling to FoxO-dependent stability. This integrated view is essential for understanding how Treg stability, metabolism, plasticity, and function are coordinately controlled.

### 3.3 Kinase-mediated control of Treg tissue trafficking and homing *in vivo*

Treg migration to peripheral tissues, a process known as homing, is essential for their immune regulatory function at sites of inflammation and tissue damage (116, 117). Homing refers to the ability of Tregs to leave the bloodstream and enter specific lymphoid or non-lymphoid tissues through interactions between homing receptors on T cells and addressing on the endothelium (118, 119). The balance between migration to lymphoid organs (via CD62L and CCR7) and to non-lymphoid tissues (via tissue-specific chemokine receptors) determines where Tregs exert their suppressive function. Kinases play a critical role in controlling Treg trafficking by regulating the expression and function of key homing molecules. The mTOR signaling pathway significantly affects the migration ability of Tregs to non-lymphoid tissues. In the Treg-specific mTOR gene knockout mouse model, mTOR deficiency results in a reduction in the migration ability of Tregs to non-lymphoid tissues, accompanied by a reduction in the proportion of Tregs in peripheral tissues. Molecular analysis shows that mTOR-deficient Tregs exhibit a characteristic phenotype of upregulated CD62L and CCR7 expression, increased S1PR1 (Sphingosine-1-phosphate receptor 1) levels and downregulated CD69 expression; these changes collectively promote the homing of Tregs to lymphoid tissues (120).

mTOR links Treg metabolic status to migration direction. mTOR blockade promotes a lymphoid tissue-homing phenotype and impairs peripheral tissue infiltration. Thus, kinase pathways do not act on isolated molecules but integrate metabolic and homing-receptor signals to coordinate Treg localization and suppressive function.

Collectively, kinases orchestrate Treg biology through multifaceted mechanisms, including metabolic signaling, Foxp3 post-translational modification, Th17/Treg balance regulation, and tissue trafficking control (**Table 2**). However, the molecular mechanisms of some kinases remain to be further elucidated. In-depth exploration of the precise regulatory network of protein kinases on Treg migration remains an important scientific challenge in the development of immunotherapeutic drugs.

## 4 Research progress in the development of Tregs-related protein kinase inhibitors

Since the approval of imatinib in 2001, protein kinase inhibitors have become an important class of therapeutic agents (121). By 2024, the FDA had approved 80 small-molecule protein kinase inhibitors for the treatment of cancers and inflammatory diseases (122). Kinase inhibitors that target Tregs work through distinct mechanisms. These can be split into direct and indirect effects on Tregs. Direct effects happen when the inhibitor binds to kinases inside Tregs, changing intrinsic signaling pathways such as PI3K/AKT/mTOR or AMPK. Indirect effects occur when the inhibitor affects other immune cells (such as Teff cells or macrophages), cytokines, or the metabolic microenvironment, which in turn alters Treg activity without directly engaging targets inside Tregs. Imatinib, Rapamycin, and their derivatives exert both direct effects on Treg-intrinsic regulation and indirect influences on the surrounding microenvironment (123, 124). Idelalisib acts primarily through direct inhibition of key signaling molecules within Tregs, thereby mediating intrinsic regulatory effects (125). In contrast, Tofacitinib acts mainly through indirect mechanisms, shaping Treg activity via effects on other immune cells, cytokines, or the metabolic microenvironment. Salicylate, a direct AMPK activator, primarily targets Tregs by modulating their metabolism, though it may also exert secondary effects through the microenvironment (126, 127). These differences provide an important theoretical foundation for the development of precise therapeutic strategies targeting Tregs.

In autoimmune diseases and chronic inflammation, Treg dysfunction lies at the core of pathogenesis, necessitating Treg enhancement to restore immune tolerance. Activation of the PI3Kδ signaling pathway helps maintain Treg function and suppresses excessive immune responses (128). Inhibition of PKC-θ has been shown to enhance Treg function and alleviate autoimmune symptoms (129). Modulating the STAT signaling pathway with JAK inhibitors such as tofacitinib may optimize the balance between Tregs and effector T cells (Teff) and improve transplant tolerance (130). In the TNBS-induced mouse colitis model, CK2 inhibitors suppress Th17 differentiation and promote Treg generation (104). CDK8/19 inhibitors promote Treg differentiation and hold potential for treating inflammatory diseases (131).

In the TME, Tregs often promote immune evasion, requiring Treg suppression to reinstate antitumor immunity. Inhibition of PI3Kδ reduces the immunosuppressive activity of Tregs and thereby enhances antitumor immunity (128). Blockade of the mTOR pathway or activation of AMPK signaling can reverse the immunosuppressive activity of Tregs, offering a new direction for cancer immunotherapy. The PI3Kδ inhibitor idelalisib reduces Treg suppressive capacity by blocking the PI3Kδ-AKT/mTOR pathway, yet continuous administration leads to intestinal Treg depletion and colitis. In contrast, intermittent dosing preserves antitumor efficacy while mitigating immune-related toxicity (125, 132). The JAK1/2 inhibitor ruxolitinib, used in the treatment of myeloproliferative neoplasms, decreases Treg numbers through inhibition of the JAK-STAT pathway, but its off-target effects increase infection risk (133).

In organ transplantation, Tregs are essential for maintaining long-term graft tolerance, calling for Treg stabilization to prevent rejection. However, in renal transplantation models, tofacitinib reduces the risk of acute rejection and preserves renal function while simultaneously increasing the incidence of Epstein-Barr virus-associated post-transplant lymphoproliferative disease (PTLD) and severe infections (134).

The effects of protein kinase inhibitors are highly context-dependent and can even produce opposing outcomes across different pathological settings, including cancer, autoimmune diseases, inflammatory conditions, and transplantation. For instance, the JAK1/3 inhibitor tofacitinib exerts potent anti-inflammatory effects in RA by stabilizing Treg function, but in renal transplantation models, it increases the risk of PTLD and severe infections (134). Similarly, inhibitors targeting TAK1, CDK8/19, and mTOR exhibit marked context dependence: they show therapeutic efficacy in inflammatory and autoimmune diseases but produce strikingly opposite effects in infectious and tumor microenvironments. At a mechanistic level, this reversal of efficacy and toxicity for a given kinase inhibitor arises from the distinct functional roles of Tregs in different disease microenvironments.

Studies on LKB1 have yielded conflicting findings: although evidence supports its role as a tumor suppressor, other studies indicate that LKB1 can also promote tumor progression by activating ROS scavenging, autophagy, and DNA repair. Consequently, no LKB1 inhibitor has yet been approved for clinical use (135). In the context of metabolic diseases and tumors, the development of AMPK modulators has progressed (136). Salicylate, a direct AMPK activator, is widely used clinically and is being explored for additional indications (137). As a key regulator of the PI3K signaling pathway, PDK1 has been the subject of multiple inhibitor patents (138). Within the PI3K/Akt/mTOR pathway, rapamycin and its derivatives (temsirolimus, everolimus) have received EMA approval as immunosuppressants and anticancer agents (139). Notably, the off-target effects of HPK1 inhibitors due to pathway crosstalk remain to be addressed (140). Current research on MAP3K9 inhibitors is primarily focused on non-coding RNA regulation (141, 142). PI3K inhibitors can be classified into pan-PI3K inhibitors, isoform-specific inhibitors, and PI3K-mTOR dual inhibitors based on their targets, each at different stages of development (143). PKA (protein kinase A), the first discovered protein kinase, requires high concentrations of chemical inhibitors (e.g., cAMP analogs, H8 and its derivatives, KT5720) to achieve efficacy, and these agents often lack specificity. Thus, novel inhibitors such as the small soluble peptide PKI are needed (144). CK2 inhibitors face challenges such as poor cell permeability (145). Although various ITK inhibitors have been developed, none have advanced to clinical research (146). TAK1, a key regulator of inflammation and cell survival, requires further optimization of its inhibitors (147). The development of STK3/4 inhibitors remains at an early stage; for example, XMU-MP-1 is associated with off-target effects (148).

Existing Treg-related protein kinase inhibitors (**Table 3**) modulate Treg activity by influencing cellular metabolism, signaling pathways, or phenotypic characteristics. Although rapamycin modulates Treg cholesterol metabolism, Treg suppressive function depends on mTORC1 activity. Thus, mTOR inhibition impairs rather than enhances Treg function. In metastatic renal cell carcinoma, the rapamycin derivative everolimus reduces absolute Treg counts and is therefore combined with low-dose cyclophosphamide, which depletes Tregs, to counter Treg-mediated immunosuppression and improve antitumor responses (149, 150). Collectively, kinase inhibitors are not universally applicable therapeutics. Their effectiveness is highly dependent on disease type, cellular microenvironment, dosing regimen, and target expression profiles. Successful clinical translation therefore requires precise context-specific matching and careful management of bidirectional risks. Owing to their multi-target properties, small-molecule kinase inhibitors hold significant therapeutic promise. Clinical studies further indicate that combining kinase inhibitors with biological agents yields better efficacy than monotherapy, likely due to target synergy.

**Table 3:** Protein kinase inhibitors targeting regulatory T cells (Tregs): mechanisms, disease contexts, and therapeutic implications.

Inhibitor name	Target (per text)	Mechanism on Treg (per text)	FDA-approved indications (per text)	Experimental animal models (per text)	Speculative therapeutic implications (per text)
Imatinib	Not specified	Direct + indirect	—	—	Modulates Treg-intrinsic regulation and the microenvironment
Rapamycin (Sirolimus) & derivatives (Everolimus, Temsirolimus)	mTOR	Direct + indirect (regulates cholesterol metabolism in Tregs; however, Treg suppressive function depends on mTORC1 signaling, so mTOR inhibition impairs Treg function)	Not FDA-approved; EMA-approved as immunosuppressants and anticancer agents (temsirolimus, everolimus)	—	Reduces absolute Treg counts (everolimus); combined with low-dose cyclophosphamide to overcome immunosuppression and enhance antitumor efficacy
Idelalisib	PI3Kδ	Direct (blocks the PI3Kδ-AKT/mTOR pathway, reduces Treg suppressive capacity)	—	—	Continuous administration leads to intestinal Treg depletion and colitis; intermittent dosing preserves antitumor efficacy while mitigating immune-related toxicity (colitis)
Tofacitinib	JAK1/3	Indirect (modulates other immune cells, cytokines, or the metabolic microenvironment; stabilizes Treg function in autoimmune settings like RA; optimizes the balance between Tregs and Teff)	—	Renal transplantation model	Improves transplant tolerance; reduces acute rejection risk and preserves renal function; however, it increases Epstein-Barr virus-associated post-transplant lymphoproliferative disease (PTLD) and severe infections
Ruxolitinib	JAK1/2	Indirect (JAK-STAT pathway inhibition, decreases Treg numbers)	—	—	Antitumor; off-target effects increase infection risk
Salicylate	AMPK (direct activator)	Direct (modulates Treg metabolic state) — may also have indirect influences via the microenvironment	None as a kinase inhibitor (widely used clinically as an anti-inflammatory, but not as a kinase inhibitor)	—	Metabolic diseases, tumors
PKC-θ inhibitor	PKC-θ	Enhances Treg function	—	—	Alleviate autoimmune symptoms
CK2 inhibitor	CK2	Suppresses Th17 differentiation and promotes Treg generation	—	TNBS-induced mouse colitis model	Treatment of inflammatory diseases (specifically colitis)
CDK8/19 inhibitor	CDK8/19	Promotes Treg differentiation	—	—	Treatment of inflammatory diseases
HPK1 inhibitor	HPK1	Off-target effects due to pathway crosstalk to be addressed	—	—	None specified
PDK1 inhibitor	PDK1	Modulates the PI3K signaling pathway	None (patents exist, but they are not FDA-approved per text)	—	None specified
TAK1 inhibitor	TAK1	Key regulator of inflammation and cell survival	—	—	Further optimization needed
STK3/4 inhibitor (e.g., XMU-MP-1)	STK3/4	Not specified in text	—	—	Associated with off-target effects
LKB1 inhibitor	LKB1	Conflicting: tumor suppressor vs. promotes tumor progression via ROS scavenging, autophagy, and DNA repair	None approved	—	None specified
PKA inhibitor (e.g., PKI)	PKA	Requires high concentrations and lacks specificity	—	—	Need for novel inhibitors
ITK inhibitor	ITK	Not specified in text	—	—	None have advanced to clinical research
MAP3K9 inhibitor	MAP3K9	Non-coding RNA regulation	—	—	Research focused on non-coding RNA regulation

A table summarizing protein kinase inhibitors that modulate regulatory T cell activity, including imatinib, rapamycin, idelalisib, tofacitinib, ruxolitinib, salicylate, and others. For each inhibitor, the table lists its kinase target, direct or indirect mechanism of action on Tregs, FDA or EMA approval status, experimental models, and speculative therapeutic implications in autoimmune diseases, cancer, and transplantation.

## 5 Conclusion and prospects

Through their immunosuppressive functions, Tregs play an essential role in immune modulation, maintenance of immune homeostasis, and the pathogenesis of autoimmune diseases and tumors. Protein kinases (PKs), which belong to the phosphotransferase family, catalyze the transfer of the γ-phosphate group from ATP to specific amino acid residues on substrate proteins, thereby mediating protein phosphorylation. As a key post-translational modification, phosphorylation is a major mechanism regulating cellular physiological activities. Recent studies have shown that various protein kinases can influence the proliferation and immunosuppressive capacity of Tregs by modulating Treg-specific molecules such as Foxp3 and CD25, as well as related signaling pathways (**Figure 2**). In turn, these effects alter immune homeostasis and ultimately regulate disease progression.

Kinases, including LKB1, AMPK, mTOR, MAP4K3 (GLK), CDK8, CDK19, PKA, ITK, NLK, STK4, CDK2, PIM1, PIM2, AKT1, Mst1, and Mst2, have been shown to influence Foxp3 expression by modulating post-translational modifications such as phosphorylation (and indirectly, methylation and ubiquitination), thereby affecting the biological functions of regulatory T cells (Tregs). Among these, AMPK, Mst1, and Mst2 can also regulate CD25 expression through the IL-2-STAT5 signaling pathway, contributing to the modulation of Treg function. Additionally, kinases such as HPK1, PDK1, and mTORC1 may impact the suppressive activity of Tregs, whereas MAP3K9 has been found to inhibit the differentiation of CD4^+^ T cells into Tregs—though the underlying mechanisms require further investigation. From the perspective of immune regulation, the dynamic balance between Th17 cells and Tregs is essential for maintaining immune homeostasis; disruption of this balance is implicated in the pathogenesis of autoimmune diseases and tumors. Existing evidence indicates that kinases such as AMPK, mTOR, PI3K, GCN2, PKA, CK2, and ITK are involved in regulating the Th17/Treg balance by influencing signaling pathways and the expression of related transcription factors. Under steady-state conditions, Tregs are primarily distributed in lymphoid tissues and the bloodstream, but they are also capable of migrating to non-lymphoid tissues and accumulating at sites of inflammation. To date, research on the molecular mechanisms by which kinases regulate Treg migration remains limited and warrants further exploration.

By integrating metabolic, transcriptional, and microenvironmental signaling pathways, protein kinases precisely govern the functional plasticity of Tregs. Targeted modulation of these kinases thus represents a promising therapeutic strategy for immune-related disorders. Current evidence has established that the regulatory mechanisms of protein kinases in Tregs provide a theoretical basis for the treatment of both tumors and autoimmune diseases, with substantial progress already achieved in the development of related inhibitors. Nevertheless, several critical challenges remain in this field: (1) the tissue-specific regulatory mechanisms and dynamic signaling balance of Tregs remain poorly understood; (2) Treg heterogeneity gives rise to distinct kinase signaling networks across different microenvironments, such as the gut and tumors; (3) the bidirectional regulatory nature of kinase signaling—exemplified by mTORC1 activation simultaneously suppressing Treg apoptosis and impairing Treg function—calls for precision intervention strategies; and (4) the off-target effects of kinase inhibitors and their impact on the Treg–effector T cell balance require urgent optimization. Future research should integrate single-cell multi-omics, kinomics, and CRISPR-based screening technologies to dissect the metabolic reprogramming of Tregs, develop microenvironment-selective inhibitors, and ultimately achieve precise control over immune activation and tolerance, thereby enhancing clinical therapeutic outcomes.

## Glossary

tTregs: thymus Tregs
nTregs: natural Tregs
pTregs: peripheral Tregs
iTregs: induced Tregs
Tregs: regulatory T cells
IL-2Rα/CD25: IL-2 receptor α chain
Foxp3: Forkhead Box P3
T1D: type 1 diabetes
SLE: systemic lupus erythematosus
RA: rheumatoid arthritis
MS: multiple sclerosis
TME: tumor microenvironment
LKB1: liver kinase B1
AMPK: AMP-activated protein kinase
PDK1: 3-phosphoinositide-dependent protein kinase 1
PI3K: phosphoinositide 3-kinase
mTOR: mammalian target of rapamycin
APC: antigen-presenting cell
DC: dendritic cell
Teff: effector T cells
Tfh: follicular helper T cells
TSDR: Treg-specific demethylated region
CNS2: conserved non-coding sequence 2
TCR: T cell receptor
GITR: Glucocorticoid-induced TNFR-related protein
CTLA-4: Cytotoxic T-Lymphocyte-Associated Protein 4
S1PR1: Sphingosine-1-phosphate receptor 1
CLA: Cutaneous lymphocyte-associated antigen
